# The Action of Obstetrical Forceps

**Published:** 1876-09

**Authors:** 


### Translations.

### THE ACTION OF OBSTETRICAL FORCEPS.

(*From the French of Prof. E. Hubert.*)

By FRED. J. HUSE, M.D.

(Continued from the July Number.)

*Resistance of the maternal tissues.* The strain of traction is exerted upon the head of the fœtus and upon the maternal organs. When traction is made exactly in the direction of the axis, and when the diameters of the head are less than those of the pelvic canal, the organs of the mother have only to sustain the friction resulting from its passage; but if the head is too voluminous to readily traverse the pelvic canal, the traction becomes transformed into lateral pressure, the resistance of the pelvis serving to compress the head. Draw a handkerchief through a ring; the resistance of the ring crowds the linen together very closely during its passage. A force of fifty kilogrammes when well directed produces, according to M. Chassagny a pressure against the walls of the pelvis of thirty kilogrammes. Tractions become decomposed, therefore, in part at least, into compressions proportional to the force exerted, and these, as my father has demonstrated, become as much greater as the departure of the traction from the direction of the axis of the strait which is to be passed.

Whether by accident or design, tractions are almost always accompanied by lateral movements. Now in giving to the forceps these movements, the obstetrician transforms it into a lever which is as much more powerful as his arm in relation to the resistance is rendered longer. The carter, says M. Chassagny, whose harness is too weak for climbing a steep hill, makes his cart turn upon one wheel, then, stopping in its turn that wheel which has progressed, he seeks by reversing the manœuvre to advance the one which previously furnished the fulcrum. But a glance of the eye upon the road is sufficient to appreciate the effect produced upon the spot which furnished the first fulcrum; in addition it is easily understood to what extent the repetition of these manœuvers might be expected to compromise the solidity of the vehicle. And he adds: lateral movements assist the accoucher in the same manner that the key assists the dentist, while they are no more favorable to mother or child than the key to the gum, alveolus or tooth. These graphic images are perhaps not entirely exact in regard to the mechanism of the lesion, but if they have the mistake of exaggerating the dangers of lateral movements, they have at least the merit of attracting attention to the new force these produce, which assists the effort of traction.

Regarding the extent to which efforts of extraction may be carried without imprudence, though it is a matter of great importance to know the exact limit, it is utterly impossible to state it precisely; certain natures resist throughout, and others yield to the slightest shock. For want of precise information we shall endeavor, in order to avoid an excess either of timidity or temerity, to give an approximate idea of the pressure which may be supported by the osseous inclosure and soft parts of the mother.

*Soft parts.* Claude Bernard has demonstrated the physiological unity of similar tissues in the animal series; the properties of the genital organs are therefore nearly the same in woman and in our larger female domestic animals. Now the veterinary surgeons frequently employ upon cows, without ever producing mortification of tissues, formidable powers: the windlass, tackle, traction of ten men, etc., and if the animal sometimes succumbs it is never when the efforts have been the most violent, but when the labor has been too prolonged. Moreover, Jobert de Lambelle, who made a special study of the vesico-vaginal fistulae of women, declares that they are invariably the consequence of too protracted delivery, never of a seasonable application of forceps.

It would appear, therefore, that the soft parts of women ought to support considerable pressure without becoming gangrenous, providing that it is not too protracted, and providing, let us add, that the vitality of the tissues is not already affected by a long labor or by dyscrasiae of diverse nature.

*Osseous parts.* In the course of his experiments, Joulin once saw the pelvic circle burst apart under a force of 288 kilogrammes, and M. Delore has tried to prove that one could pull with a force of 200 kilogrammes without danger of producing any rupture. We are acquainted with a case of pubic diastasis produced by the traction of an obstetrician simply pulling upon the forceps, and it is not so very wonderful, since, in cases which necessitate employment of much force, the bones have suffered either from rachitis or mollites ossium, and they no longer possess the normal consistence. Thus, it is not allowable, in regard to the osseous parts, to make use of the amount of force which the soft parts might appear to be able, in certain circumstances, to support without excess of danger.

*Muscular force.* Each obstetrician should know with exactness the force which he is capable of exerting by means of the forceps, since the mother and child are to sustain the result. The following are the averages furnished by three generations of our pupils pulling upon the cords attached to a small dynameter of Matthieu: they are higher than those indicated by Joulin and M. Delore: as said Julius Cæsar, *fortissimi belgœ* :

*One man*—pulling solely with the arm, without fulcrum, 65 kilogrammes; the foot taking support at the height of the instrument, 147 kilogrammes.

*Two men*—pulling without fulcrum, 110 kilogrammes; with fulcrum upon the floor, 240 kilogrammes.

Adding to these figures as much as is really added by giving the instrument lateral movement, it will be seen that by using two hands upon the forceps there may be obtained an array of force quite appalling.

Muscular force, though excellent for overcoming slight resistance, becomes of less use in surmounting those more serious. In struggling against a considerable obstacle our efforts become sudden, abrupt, jerking; like the mired horse, we strain every muscle. The muscles contract forcibly for several seconds, then relax, and their energy, at the outset most vigorous, wanes rapidly towards extinction in the fatigue at the end of a short series of interrupted efforts that become less and less powerful. It is evident that tractions of this sort, violent, sudden, jerking, can neither be favorable to the head of the child, nor the tissues of the mother, nor the engagement of a rounded tumor in an elliptical canal, and that a regular mechanical force which can be measured by the dynameter and sustained is far preferable. Joulin has shown experimentally that in those instances where manual force developed by two men was sufficient to engage the head of the fœtus in a given opening, a sustained mechanical traction produced the same result with far less force. Now this is a fact of extreme importance, in our opinion, for to economize force is, for mother and child alike, to economize compression.*

The ear is no more satisfied by a scale, the last note of which is not given, than is the mind by a series of propositions lacking a conclusion. We shall therefore give the note of the octave and conclude.

We are opposed to placing the forceps upon the head of an infant at term retained above a strait of less than seventy-five millimeters. This would be followed by one of two things: either the crushing of the head or breaking apart the pelvis, for the bi-auricular cranial diameter measures seventy-five millimeters and is irreducible.

With the pelvis offering at least a space of seventy-five millimeters between the points most nearly approximated, the head can pass with rigor, but upon the condition that the cranial vault changes form, and, when it measures nine centimeters, that the reduction should be about one and one-half centimeters. Nature,† as we have said, often accomplishes this

* "Whatever," says Joulin, "may be the prejudice against novelties, there is yet a result well worthy to arrest attention: it is that the 'aid forceps' extracts the fœtus with an expenditure of force less by 30, 40 or 50 kilogrammes than that necessary in manual tractions, which nevertheless failed completely in instances where the instrument succeeded." The superiority of continued traction, compared with intermitting traction, has been demonstrated by calculus and by the experiments of M. Marcy.

† Among others, we have seen two children born under these conditions: one had facial hemiplegia, with the other the pressure of the sacro-vertibral angle had produced a limited gangrene of the scalp, only cured three weeks after birth.

reduction without killing the child, and when she is disposed to undertake it, she should be let alone, since she will do better than we. But the instances where the uterine forces triumph happily over such obstacles are exceptional, and generally art is obliged to come to the rescue of the woman in peril.

Let us give a rapid glance at the resources at its disposal. Certain authors have advised version; but this is to substitute for the difficulty an operation which is not without gravity. Prolonged waiting and ordinary forceps used with energetic muscular tractions, deeply compromise the child and are not without danger to the mother. Gastrotomy is an extreme measure to which the obstetrician only resorts in the most urgent cases; and, regarding symphseotomy, it is nearly of equal danger, so that the woman has the incontestable right to decline the heroic self-renunciation of a consent to the risks of either operation. Less perilous to the mother than these two operations, embryotomy is none the less open to serious objections; its legitimacy is debatable; moreover it is sometimes fatal to the mother, and such being the fact, what woman would authorize the attempt, or what practitioner would dare to propose it, if art presented a resource for the occasion which was less perilous for the mother and was not surely fatal to the child?

The Flemish lever * is perhaps the most suitable instrument in difficult circumstances which can be obtained. Where the pelvic diameter is too short it can be applied upon the corresponding cranial diameter, but it requires a prudent and skillful hand. Still nearer the reach of the generality of physicians than the lever, and appearing to us of all means proposed the least perilous to the child, is careful mechanical traction of a moderate degree, regulated by a dynameter. It accomplishes the maximum of result with the minimum expenditure of force, and consequently affords a happy improvement upon manual traction, which is accomplished by more or less violence.

We are convinced that when there is prepared a comparative statement of labors terminated by mechanical force and

* Lever of the third class.

those in which manual force is used, in pelves of 75, 80 and 85 millimeters, the result will confirm theoretical assumptions, and that then — in spite of certain authorities deterred by routine, yet unable to prevail against reason and experience— the stubborn, violent traction of one obstetrician, and *a fortiori* of two, upon the forceps will be abandoned for means at once more efficacious and more gentle. *Non vi, sed arte.*

We have on two occasions applied the aid-forceps of Joulin,* once in a pelvis which we had estimated at 76 millimeters, but which really only measured 73; the other time in a pelvis of 75 millimeters. We terminated the labor with a force of 40 kilogrammes — that is to say, a force one-half less than if we had pulled with our arms — on one occasion in an hour, on the other in twenty-two minutes. The mothers made no complaint against the action of the apparatus and had most prosperous confinements. The infants could not be resuscitated; though the heart was still pulsating in both of them, the nervous centers had suffered so severely that respiration could not be established. Without entering into any criticism upon the imperfections in traction apparatus which time will doubtless remove, there is a general statement which we think should be made at this point. Continuity seems to us to be an unfavorable condition of mechanical traction, alike for the maternal tissues and the head of the child. Nature does not thus exert its forces without intermission; each pain is followed by an interval of repose, a lull during which the circulation which has just been much embarrassed if not interrupted, may resume its course. In future, when employing mechanical apparatus, we shall take care, at the risk of slightly prolonging the operation, to detach the cords from time to time.

* Bulletin de l'Acad. royale de méd. de Belgique, T. IV., 3e série, no. 3.

